# First identification and genotyping of *Enterocytozoon bieneusi* in humans in Myanmar

**DOI:** 10.1186/s12866-019-1694-1

**Published:** 2020-01-13

**Authors:** Yujuan Shen, Baiyan Gong, Xiaohua Liu, Yanchen Wu, Fengkun Yang, Jie Xu, Xiaofan Zhang, Jianping Cao, Aiqin Liu

**Affiliations:** 1 National Institute of Parasitic Diseases, Chinese Center for Disease Control and Prevention, Chinese Center for Tropical Diseases Research, WHO Collaborating Center for Tropical Diseases, Key Laboratory of Parasite and Vector Biology, MOH, Shanghai, 200025 China; 2 Department of Parasitology, Harbin Medical University, Harbin, 150081 Heilongjiang China

**Keywords:** *Enterocytozoon bieneusi*, Humans, Prevalence, Genotype, Phylogeny

## Abstract

**Background:**

Intestinal pathogen infections are widespread among impoverished populations. *Enterocytozoon bieneusi* is the most common pathogen of intestinal microsporidian species in humans worldwide*.* However, no epidemiological information is available on *E. bieneusi* infection in humans in Myanmar. The present study comprised the first identification and genotyping of *E. bieneusi* in humans conducted in Myanmar.

**Results:**

A total of 172 fecal specimens were collected from the Wa people (one each) in four villages of Pangsang Township of the Matman District of Shan State, Myanmar, and each participant completed a questionnaire. *E. bieneusi* was identified and genotyped using polymerase chain reaction (PCR) and sequence analysis of the internal transcribed spacer (ITS) region of the ribosomal RNA (rRNA) gene. The average prevalence of *E. bieneusi* was 8.72% (15/172), ranging from 3.85 to 13.89% by village. *E. bieneusi* infection was not related to any of the risk factors studied. Six genotypes were identified, comprising two known genotypes Peru6 (*n* = 10) and D (*n* = 1) and four novel genotypes (MMR23, MMR25, MMR86, and MMR87) (one each), and two people infected with genotype Peru6 were from the same family. A phylogenetic analysis based on a neighbor-joining tree of the ITS sequences of *E. bieneusi* indicated that all the six genotypes were clustered into group 1.

**Conclusions:**

This is the first identification and genotyping of *E. bieneusi* in humans in Myanmar. The observations that the two people infected with genotype Peru6 were from the same family, and that all six genotypes obtained in the present study fell into zoonotic group 1, showed the potential for anthropogenic and zoonotic transmissions. The present data argue for the importance of epidemiological control and prevention from medical sectors.

## Background

Microsporidia comprise a diverse group of unicellular and obligate intracellular parasitic fungi and have been found in a wide range of vertebrate and invertebrate hosts [1]. To date, there are about 1500 species in over 200 genera formally described, and 17 species have been found in humans with *Enterocytozoon bieneusi* being the most frequently diagnosed (over 90%) in cases of microsporidioses [2, 3]. *E. bieneusi* is one of the most common intestinal pathogens responsible for diarrhea in infected hosts, and more seriously, chronic or life-threatening diarrhea often occur in immunodeficient or immunocompromised individuals, such as patients with acquired immune deficiency syndrome (AIDS), organ transplant recipients, patients with cancer, travelers, children, and the elderly, showing the clinical features of opportunistic infection [4]. The increasing number of cases of asymptomatic infection of *E. bieneusi* in immunocompetent people highlights the epidemiological importance and significance that they play in the transmission of the microorganism [5]. The identification of *E. bieneusi* in numerous mammal and bird species indicates the zoonotic nature of this disease [6]. An unusual genotype, Peru16, was found in seven guinea pigs and a 2-year-old child in the same household [7]. Of course, humans could also acquire *E. bieneusi* infections through the anthroponotic route. Two studies conducted in a Thai orphanage provided evidence of person-to-person transmission of *E. bieneusi*, based on the findings that genotype A was detected in all *E. bieneusi*-positive fecal samples of the orphans [8, 9].

However, most human infections of *E. bieneusi* are considered to result from fecal-oral transmission of infective spores from infected hosts through contaminated food or water [3]. *E. bieneusi* has been detected in some foods (milk, raspberries, beans, and lettuce) [10, 11] and a foodborne outbreak caused by *E. bieneusi* was reported in Sweden in 2009 [11]. To date, no waterborne outbreaks have been reported. However, *E. bieneusi* spores have been detected in multiple water supplies, including irrigation waters used for crops, recreational waters, and effluents from wastewater treatment plants [2]. Contact with contaminated water is considered an important risk factor related to *E. bieneusi* infection in epidemiological studies [4].

**Fig. 1:**
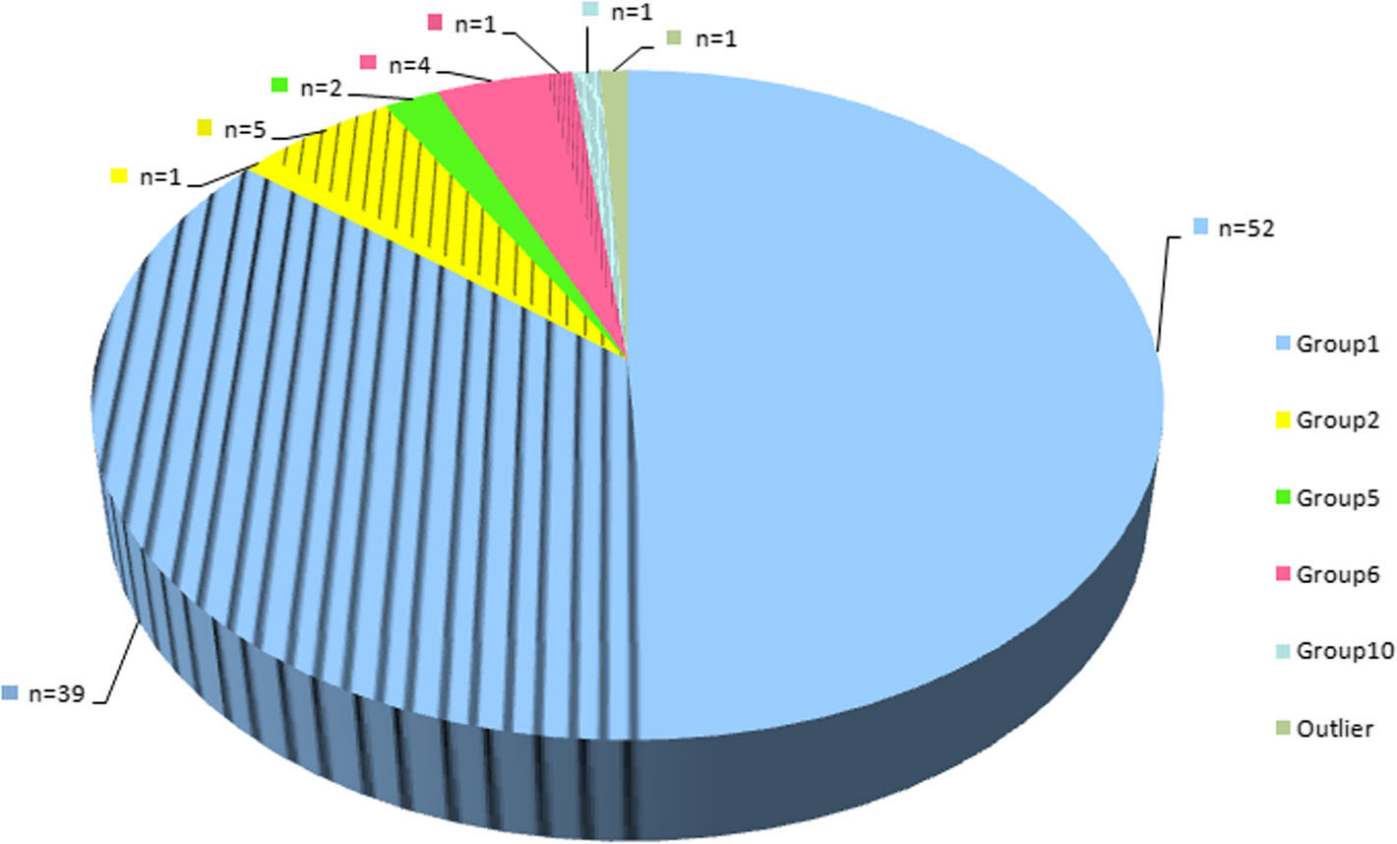
Distribution of *E. bieneusi* genotypes in humans by group worldwide (*n* = 106). Shaded parts represent the genotypes in both humans and animals. The data presented here are based on the work of Li et al. [2]

*E. bieneusi* is a complex species with multiple genotypes. By sequence analysis of the internal transcribed spacer (ITS) region of the ribosomal RNA (rRNA) gene, at least 474 ITS genotypes have been identified, which can be phylogenetically divided into 11 groups (groups 1–11). Groups 1 and 2 are two large groups, composed of 314 and 94 genotypes, respectively [2]. To date, at least 106 genotypes have been identified in humans**:** 91 in group 1, six in group 2, two in group 5, five in group 6, one in group 10, and one in the outlier, and 46 of them have been found in animals**:** 39 in group 1, five in group 2, one in group 6, and one in group 10 (Fig. 1). The genotypes in group 1 originate from a wide host range, including humans and numerous mammal and bird species, revealing low host specificity and the potential for zoonotic or cross-species transmission [2]. The genotypes in group 2 were once considered cattle-specific based on early studies [12]. However, with increasing epidemiological data for *E. bieneusi*, it has been observed that the genotypes in this group could be detected in other animal hosts; more importantly, certain genotypes (notably BEB4, BEB6, CHN3, I and J) have been found in humans, raising public health concern related to the zoonotic potential of this group [2].

Infectious diseases, including microsporidioses, usually affect people living in poverty, and these diseases may further promote poverty. Intestinal pathogen infections are widespread among Southeast Asia’s most impoverished populations [13]. In Myanmar, *Cryptosporidium*, *Giardia duodenalis*, *Entamoeba histolytica*, *Entamoeba coli*, and *Entamoeba nana* have been detected in humans [14, 15]. However, no data are available on *E. bieneusi* infection in humans in this country. The Wa is one of the 135 officially recognized ethnic groups of Myanmar. Wa people live mostly in small villages in Shan State, close to the Chinese border, where the economy and culture are undeveloped, and villagers do not practice good hygiene habits (e.g., washing hands before eating meals). The objectives of the present study were to understand the prevalence and genotypes of *E. bieneusi* in humans in Myanmar, specifically in Wa people, using polymerase chain reaction (PCR) and sequence analysis of the ITS region of the rRNA gene as well as possible risk factors related to *E. bieneusi* infection.

## Results

### Prevalence of *E. bieneusi*

A total of 172 fecal specimens were collected and studied for the presence of *E. bieneusi* using PCR amplification of the ITS region of the rRNA gene. A total of 15 PCR products of the expected size were confirmed positive for *E. bieneusi* by sequence analysis. The overall prevalence of *E. bieneusi* was 8.72% (15/172) in Wa people in the investigated areas. *E. bieneusi* was found in all the four investigated villages, with a prevalence ranging from 3.85 to 13.89%. However, there was no statistically difference among them according to a χ^2^ test (*P* > 0.05) (Table 1).

**Table 1:** Prevalence and distribution of *E. bieneusi* genotypes in humans by village

Collection site	Positive no./Examined no. (%)	Genotype/s (*n*)^a^
Village I	4/32 (12.50)	Peru6 (1), D (1), **MMR23 (1), MMR25 (1)**
Village II	2/52 (3.85)	Peru6 (2)
Village III	4/52 (7.69)	Peru6 (2), **MMR86 (1), MMR87 (1)**
Village IV	5/36 (13.89)	Peru6 (5)
Total	15/172 (8.72)	Peru6 (10), D (1), **MMR23 (1), MMR25 (1), MMR86 (1), MMR87 (1)**

^a^ The genotypes in bold are novel genotypes obtained in the present study

### Relationship of *E. bieneusi* infection and risk factors as well as gastrointestinal symptoms

**Fig. 2:**
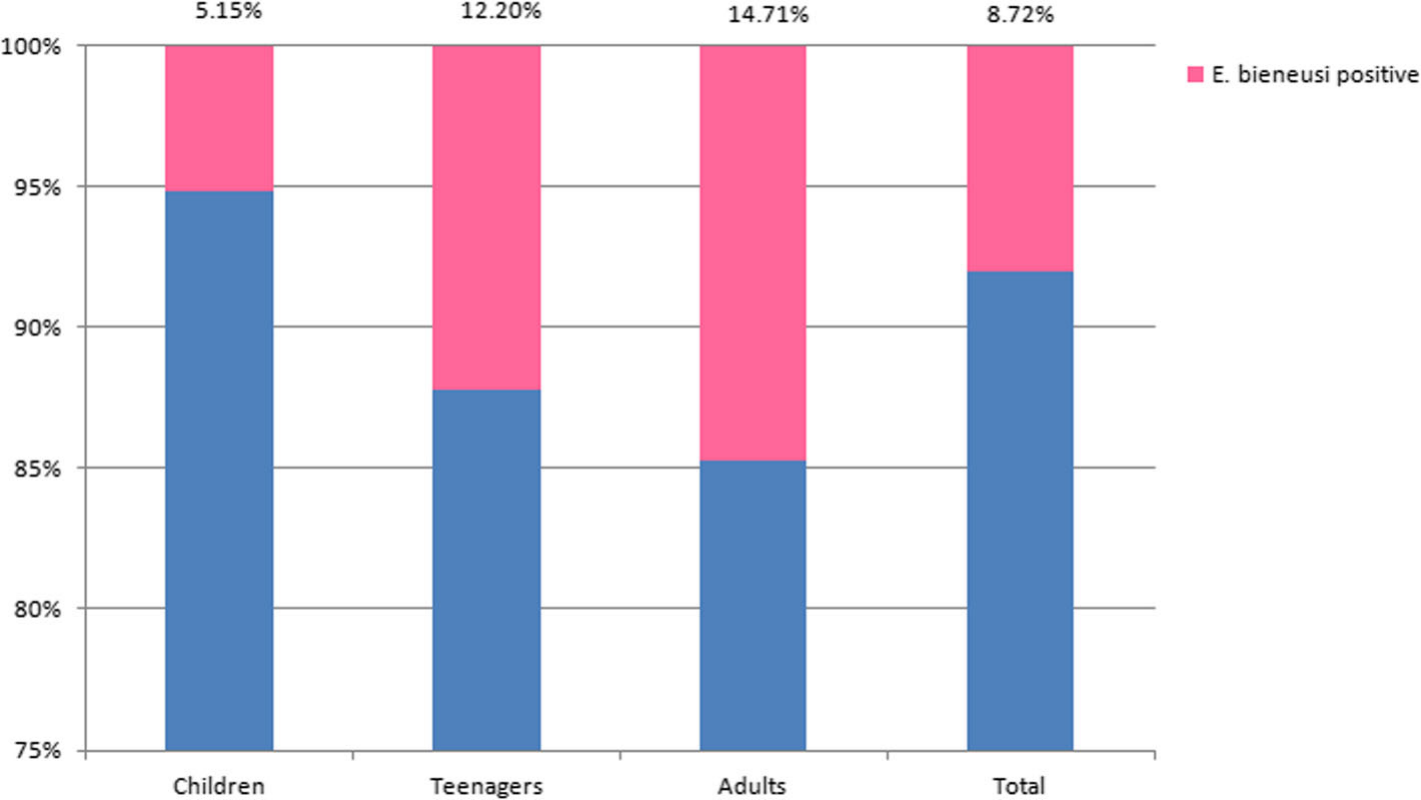
Percentage of *E. bieneusi* infection in humans by age

In the present study, with the help of the local Center for Disease Control and Prevention (CDC) and the people at Health Without Borders, all the participants, including 97 children (aged 7–12 years), 41 teenagers (aged 13–17 years), and 34 adults (aged > 18 years), responded to the questionnaires after they received an oral explanation of the study objectives and procedures, and provided complete information in writing. There were no significant relationships between *E. bieneusi* infection and each of the risk factors listed in Table 2 and Fig. 2. Meanwhile, the presence of *E. bieneusi* in fecal specimens was not related to gastrointestinal symptoms listed in Table 2. Similar results were observed about relationship of *E. bieneusi* infection and risk factors, as well as gastrointestinal symptoms, in the largest age group of children (Table 3).

**Table 2:** Analysis of risk factors for *E. bieneusi* infection

Variable			Examined no. (%)	Positive no (%)	OR^a^ (95% CI^b^)	χ2/*P*-value
Demographic factor	Sex	Male	94/54.65	11/11.70	2.45 (0.75, 8.03)	1.56/0.21
		Female	78/45.35	4/5.13		
	Age (years)	Children (< 13)	97/56.39	5/5.15	Ref	
		Teenagers (13–17)	41/23.84	5/12.20	0.39 (0.11, 1.43)	2.13/0.15
		Adults (≥ 18 years)	34/19.77	5/14.71	0.32 (0.09, 1.17)	3.26/0.07
Clinical symptoms	Diarrhea	Yes	42/24.42	3/7.14	0.76 (0.20, 2.82)	0.01/0.92
		No	130/75.58	12/9.23		
	Abdominal pain	Yes	24/13.95	2/8.33	0.94 (0.20, 4.47)	0/1.00
		No	148/86.05	13/8.78		
	Nausea	Yes	5/2.91	0	1.10 (1.05, 1.15)	−/1.00^c^
		No	167/97.09	15/8.98		
	Emesis	Yes	8/4.65	1/12.5	1.53 (0.18, 13.35)	0/1.00
		No	164/95.35	14/8.54		
	Anorexia	Yes	6/3.49	1/16.67	2.17 (0.24, 19.91)	0/1.00
		No	166/96.51	14/8.43		
Personal hygiene habits	Drinking boiled water	Yes	135/78.49	12/8.89	1.11 (0.30, 4.14)	0/1.00
		No	37/21.51	3/8.11		
	Washing hands before meals	Yes	98/56.98	9/9.18	1.15 (0.39, 3.38)	0.06/0.80
		No	74/43.02	6/8.11		
	Washing hands after using toilets	Yes	73/42.44	9/12.33	2.18 (0.74, 6.42)	2.07/0.15
		No	99/57.56	6/6.06		
	Eating unwashed vegetables and fruits	Yes	145/84.30	15/10.34	0.90 (0.85, 0.95)	−/0.13^c^
		No	27/15.70	0	Ref	
Others	Pit toilets	Public	166/96.51	15/9.04	0.91 (0.87, 0.95)	−/1.00^c^
		Individual	6/3.49	0		
	Animal feeding patterns	Free-ranging	36/20.93	2/5.56	0.56 (0.07, 4.29)	0/0.98
		Both free-ranging and captive	115/66.86	11/9.57	1.01 (0.21, 4.90)	0/1.00
		Captive	21/12.21	2/9.52	Ref	

^a^ *OR* Odds ratio. ^b^ *CI* Confidence interval. ^c^ Fisher’s exact test

**Table 3:** Analysis of risk factors for *E. bieneusi* infection in children

Variable	Examined no. (%)	Positive no (%)	OR^a^ (95% CI^b^)	χ2/*P*-value
Demographic factor				
Sex				
Male	44/45.36	4/9.09	5.20 (0.56, 48.35)	1.29/0.26
Female	53/54.64	1/1.89		
Clinical symptoms				
Diarrhea				
Yes	26/26.80	2/7.69	1.89 (0.30, 12.00)	0.03/0.87
No	71/73.20	3/4.23		
Abdominal pain				
Yes	14/14.43	1/7.14	0.66 (0.07, 6.36)	0/1.00
No	83/85.57	4/4.82		
Nausea				
Yes	3/3.09	0	1.03 (1.00, 1.07)	−/1.00^c^
No	94/96.91	5/5.32		
Emesis				
Yes	2/2.06	0	1.02 (1.00, 1.05)	−/1.00^c^
No	95/97.94	5/5.26		
Anorexia				
Yes	2/2.06	0	1.02 (1.00, 1.05)	−/1.00^c^
No	95/97.94	5/5.26		
Personal hygiene habits				
Drinking boiled water				
Yes	71/73.20	2/2.82	4.50 (0.71, 28.63)	1.45/0.23
No	26/26.80	3/11.54		
Washing hands before meals				
Yes	42/43.30	4/9.52	0.18 (0.02, 1.64)	1.53/0.22
No	55/56.70	1/1.82		
Washing hands after using toilets				
Yes	31/31.96	0	1.08 (1.01, 1.16)	−/0.17^c^
No	66/68.04	5/7.58		
Eating unwashed vegetables and fruits				
Yes	79/81.44	5/6.33	1.24 (1.12, 1.38)	−/0.58^c^
No	18/18.56	0		
Others				
Pit toilets				
Public	95/97.94	5/5.26	1.02 (1.00, 1.05)	−/1.00^c^
Individual	2/2.06	0		
Animal feeding patterns				
Free-ranging	29/29.90	1/3.45	0.39 (0.02, 6.85)	0/1.00
Both free-ranging and captive	56/57.73	3/5.36	0.62 (0.06, 6.56)	0/1.00
Captive	12/12.37	1/8.33	Ref	

^a^ *OR* Odds ratio. ^b^ *CI* Confidence interval. ^c^ Fisher’s exact test

### Genetic characterizations and genotype distribution of *E. bieneusi* by village

By multiple-sequence alignment and analysis of the ITS region of the rRNA gene, 18 polymorphic sites were observed among 15 *E. bieneusi* isolates obtained in the present study (Table 4). Six genotypes were identified, including two known genotypes Peru6 (*n* = 10) and D (*n* = 1) and four novel genotypes (one each) named as MMR23 (GenBank: MN399816), MMR25 (GenBank: MN399817), MMR86 (GenBank: MN399818) and MMR87 (GenBank: MN399819), which all had the largest similarity with the known genotypes Peru6 (KY950540) (99.59%), Peru6 (KY950540) (98.77%), GDR2 (MH714715) (99.18%) and Henan-II (JF691565) (99.59%), respectively. The dominant genotype, Peru6 (66.67%, 10/15), could be found in all four villages. Meanwhile, two of ten people infected with genotype Peru6 were from the same family.

**Table 4:** Variation at 18 polymorphic sites within the ITS sequences of *E. bieneusi* isolates in Myanmar

Genotype (no.)	GenBank accession no.	Nucleotide at position:																	
		12	30	31	35	38	58	81	93	100	113	117	131	138	169	176	195	200	221
Known^a^																			
Peru6 (10)	KY950540	G	T	A	T	A	A	C	T	A	C	G	G	G	G	A	G	A	A
D (1)	MG491314	G	T	G	T	G	G	C	C	G	C	T	G	G	G	A	G	A	A
Novel																			
MMR23 (1)	MN399816	G	T	A	T	A	A	C	T	A	C	G	G	G	A	A	G	A	A
MMR25 (1)	MN399817	G	T	A	T	G	A	C	T	A	C	G	G	G	A	A	A	A	A
MMR86 (1)	MN399818	T	C	A	C	G	G	T	T	G	T	T	A	A	G	G	G	A	A
MMR87 (1)	MN399819	G	T	G	T	G	G	C	C	G	C	T	G	G	G	A	G	G	G

^a^ Accession numbers of known genotypes, indicating that *E. bieneusi* isolates have 100% homology with the sequences from GenBank

### Phylogenetic relationship of *E. bieneusi* genotypes

**Fig. 3:**
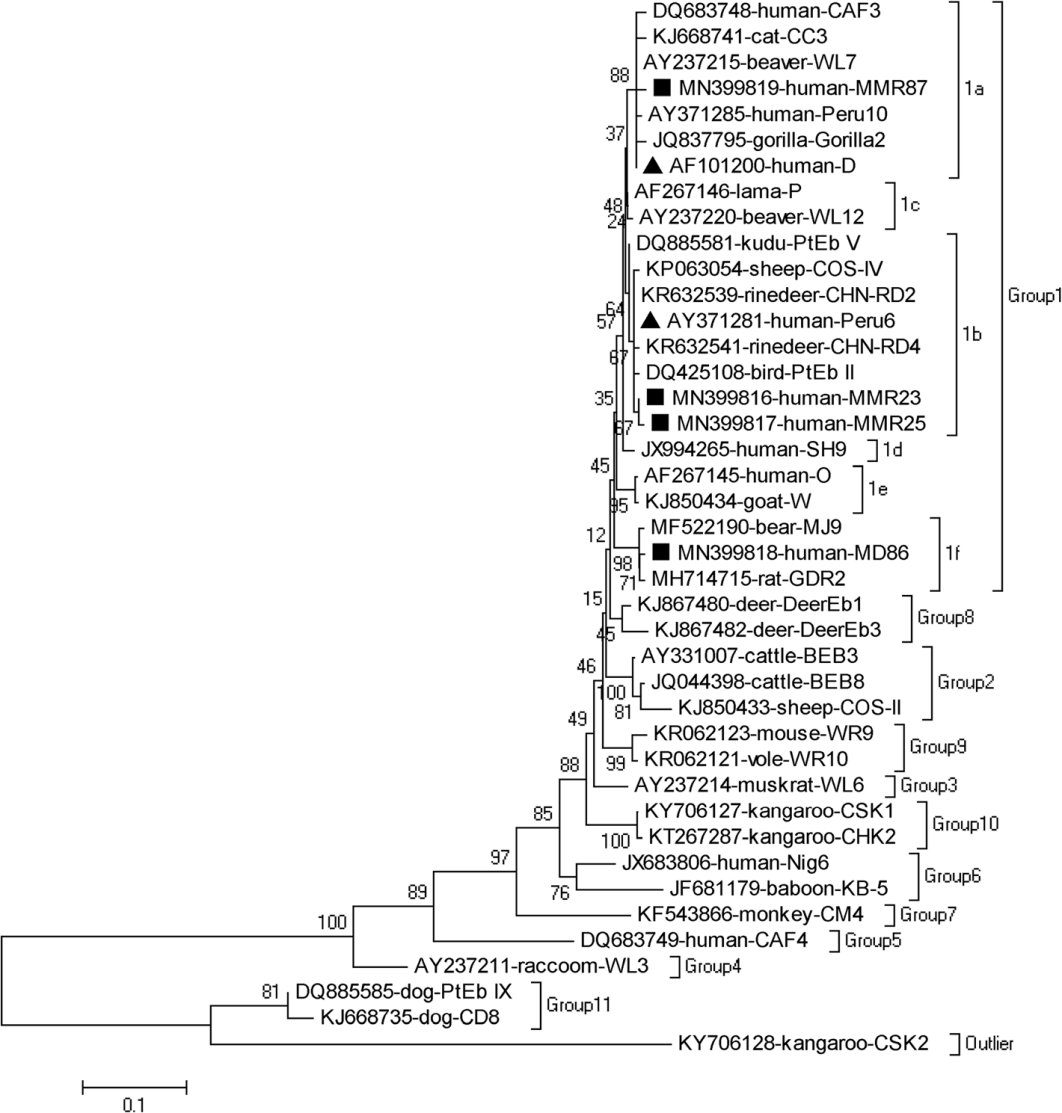
Phylogenetic relationships of the genotypes of *E. bieneusi*. The relationships of the genotypes of *E. bieneusi* identified in this study and known genotypes published in GenBank were inferred using a neighbor-joining analysis of ITS sequences based on genetic distances calculated by the Kimura 2-parameter model. The numbers on the branches are percent bootstrapping values from 1000 replicates. Each sequence is identified by its accession number, host origin, and genotype designation. The group terminology for the clusters is based on the work of Li et al. [2]. The triangles and squares filled in black indicate known genotypes and novel genotypes identified in this study, respectively

In a phylogenetic analysis of the ITS sequences of *E. bieneusi*, the genetic relationship was observed among *E. bieneusi* genotypes identified in the present study. All six genotypes obtained here were clustered into zoonotic group 1: genotypes Peru6, MMR23, and MMR25 in subgroup 1b; genotypes D and MMR87 in subgroup 1a; and genotype MMR86 in subgroup 1f (Fig. 3).

## Discussion

Intestinal pathogens are considered to be prevalent in areas with poor sanitation and low socioeconomic status [15]. Meanwhile, higher temperatures often increase parasite growth, reproduction and infectivity [16, 17]. In Myanmar, there is a high annual average temperature (21–32 °C), resulting in the widespread presence of intestinal pathogens [15]. In the present molecular epidemiological investigation of *E. bieneusi* conducted in 172 Wa people from four villages of Pangsang Township of Matman District of Shan State, 8.72% of villagers were confirmed to be infected with *E. bieneusi*, with a prevalence ranging from 3.85 to 13.89% by village. The result was similar to that of a recent epidemiological study of *E. bieneusi* conducted in villagers in Yunnan, China, bordering with Myanmar, in which the average prevalence of *E. bieneusi* was 8.30% (24/289) in Yao people [5]. This similar prevalence might be related to similar climatic and socio-economic conditions on both sides of the China–Myanmar border. In fact, many factors might have influence on *E. bieneusi* infection in humans.

Immunodeficient or immunocompromised individuals, especially those associated with human immunodeficiency virus (HIV) infection and AIDS, are susceptible to infection with *E. bieneusi*. *E. bieneusi* has been reported to be more common in HIV-positive patients than in HIV-negative patients, such as 5.0% versus 1.7% in India [18] and 7.0% versus 5.1% in Portugal [19]. A high prevalence of *E. bieneusi* has also been found in HIV-positive patients, such as 27.3% in Thailand, 11.58% in China and 9.5% in India [20–22]. Children have been identified as a population group at risk for *E. bieneusi* infection because of immature immune system and lack of good hygienic habits [19]. A high prevalence of *E. bieneusi* has been reported in non-diarrheal children (9.3%) in Nigeria [23], and in diarrheal children (22.5%) in China [24]. In Uganda, HIV-positive children were observed to be more likely to have *E. bieneusi* in their stool than HIV-negative children (76.9% versus 6.6%) [25]. Some studies reported a high prevalence of *E. bieneusi* in elderly persons (17.0%) and in cancer patients (40%) [26, 27]. In addition, poor personal hygiene habits possibly increase the risk of *E*. *bieneusi* infection. In China, the HIV/AIDS patients who drink unboiled water and/or do not wash hands before meals showed a statistically higher prevalence of *E. bieneusi* than the other patients (*P* < 0.05) [22, 28]. However, in the present study, no correlation was found between *E. bieneusi* infection and any of risk factors analyzed. Similarly, statistical calculations showed that none of the gastrointestinal symptoms recorded was related to *E. bieneusi* infection. Reported symptoms might be caused by other intestinal pathogens. Indeed, the appearance and severity of clinical symptoms are affected by multiple factors. In addition to the immune status of the infected hosts, intestinal pathogen species and their genetic background as well as the infection intensity in humans, could make differences in clinical symptoms. Even changes in diet can affect fecal consistency (e.g., the presence or absence of diarrhea). Therefore, future work will focus on the relationship between *E. bieneusi* infection and clinical symptoms, and the interaction of intestinal pathogens, including *E. bieneusi* in clinical gastrointestinal symptoms.

In the present study, based on sequence analysis of the ITS region, 18 polymorphic sites were observed among 15 *E. bieneusi* isolates and six genotypes were identified: two known genotypes (Peru6 and D) and four novel genotypes (named as MMR23, MMR25, MMR86 and MMR87). Genotype Peru6 showed an absolute predominance (66.67%, 10/15) in Wa people in the investigated areas, similar to a recent study conducted in neighboring China (87.50%, 21/24) [5]. Genotype Peru6 is actually a rare genotype in humans. Since the first identification in humans in Peru in 2003 [29], this genotype has only been found in humans in Peru [7, 30, 31], Portugal [19] and China [5]. Genotype D is the most prevalent genotype detected in humans. To date, this genotype has been found in humans in four continents, including Asia (China, India, Iran, Thailand and Vietnam), South America (Brazil and Peru), Europe (Netherlands, Poland, Portugal, Russia, Spain and UK) and Africa (Cameroon, Congo, Gabon, Malawi, Niger, Nigeria, Sao Tome and Tunis) [2]. Both genotypes Peru6 and D have a wide host range of animals: Peru6 in eight and nine species of mammals and birds [5], respectively, and D in 24 and seven species of mammals and birds [2], respectively, indicating the possibility of zoonotic transmissions. In a phylogenetic analysis of the ITS sequences of *E. bieneusi*, the observation that all four novel genotypes (MMR23, MMR25, MMR86 and MMR87) fell into group 1 suggests the possibility of zoonotic transmission. In addition, among 10 people infected with genotype Peru6, two of them were confined to the same family, indicating the possibility of anthropogenic transmission.

The present study also has some limitations. The sample size of this ethnic group in Myanmar was very small; therefore, these results might not reflect the prevalence of *E. bieneusi* in humans in this country. The true burden of human microsporidioses caused by *E. bieneusi* needs to be assessed by systematic molecular epidemiological investigations in the future.

## Conclusions

The present study is the first identification and genotyping of *E. bieneusi* in humans in Myanmar. An average prevalence of *E. bieneusi* was 8.72% (15/172) in Wa people in the investigated areas, and six genotypes were identified, with genotype Peru6 being dominant. The previous observation of genotypes Peru6 and D in numerous animals, and the fact of four novel genotypes (MMR23, MMR25, MMR86 and MMR87) falling into group 1 suggest the possibility of zoonotic transmission. Thus, it is necessary to carry out epidemiological investigations of *E. bieneusi* in local animals. This is an important step in adequately controlling *E. bieneusi* infection in humans, particularly in the absence of an effective vaccine and available drugs. Although fumagillin has been used successfully in humans to treat intestinal microsporidioses caused by *E. bieneusi*, its efficacy is counterbalanced by its adverse effects [32]. The possible existence of anthropogenic transmission highlights the importance of health education in people living in the investigated areas. The present data argue for the importance of epidemiological control and prevention from medical sectors.

## Methods

### Study population

In October, 2018, fresh fecal specimens (approximately 5–10 g) were collected from 172 Wa people aged from 7 to 53 years distributing in four villages of Pangsang Township of Matman District of Shan State, Myanmar (geographical coordinates: 99.11 °E longitude, 22.10 °N latitude). All the fecal specimens were from immunocompetent individuals. Only one specimen per participant was included in the present study. Information about participants was collected and recorded by a structured questionnaire including socio-demographic characteristics, possible risk factors related to *E. bieneusi* infection as well as common gastrointestinal symptoms. The specimens were transported to the laboratory in a cooler with ice packs within 24 h after collection and stored in refrigerators at − 20 °C before DNA extraction.

### DNA extraction

Genomic DNA of *E. bieneusi* was directly extracted from 180 to 200 mg of each of 172 fecal specimens using a QIAamp DNA stool mini kit (QIAgen 51,504, Hilden, Germany) according to the manufacturer’s instructions. All the reagents were provided by the manufacturer. To obtain a high yield of DNA, the lysis temperature was increased to 95 °C. DNA was finally eluted in 200 μl of AE and stored at − 20 °C in a freezer prior to PCR analysis.

### PCR amplification.

*E. bieneusi* was identified and genotyped by PCR of an approximately 410-bp of the rRNA gene that covered the entire ITS region (243 bp) using nested primers as described [33]. TaKaRa Taq DNA Polymerase (TaKaRa Bio Inc., Tokyo, Japan) was used for all PCR amplifications. A positive control (DNA of a sheep-derived genotype COS-IV) and a negative control (no DNA water) were included with each batch of specimens analyzed. Each specimen was analyzed at least twice. All secondary PCR products were subjected to electrophoresis in a 1.5% agarose gel and visualized under UV by staining the gel with GelStrain (TransGen Biotech., Beijing, China). Details on methods are available in Protocols.io open access repository at the following link: dx.doi.org/10.17504/protocols.io.yvjfw4n.

### Nucleotide sequencing and analyzing

All secondary PCR products of the anticipated size were sent to Comate Bioscience Company Limited (Jilin, China) for DNA sequencing on an ABI Prism 3730 XL DNA Analyzer by Sinogeno-max Biotechnology Co., Ltd. (Beijing, China), using the Big Dye Terminator v3.1 Cycle Sequencing Kit (Applied Biosystems, USA). The sequence accuracy was confirmed by two-directional sequencing and by sequencing a new PCR product if necessary. Nucleotide sequences obtained in the present study were aligned with each other and reference sequences deposited in the GenBank database using the Sequence Basic Local Alignment Search Tool (BLAST) and Clustal X 1.83 (http://www.clustal.org/). All the genotypes were identified only based on 243 bp of the ITS region of the rRNA gene of *E. bieneusi* according to the established nomenclature system [34]. The first published names of the genotypes would be given if they produced sequences identical to those previously deposited in the GenBank database. In contrast, novel genotypes producing different sequences from published ones were given genotype names by adding specimen codes behind MMR (the abbreviation of Myanmar).

### Phylogenetic and statistical analyses

The genetic proximity of all the genotypes obtained in our study was compared with each other and previously reported genotypes by constructing a neighbor-joining analysis of the ITS sequences based on genetic distances calculated by the Kimura two-parameter model implemented in the program Mega 5 (http://www.megasoftware.net/). A bootstrap analysis with 1000 replicates was used to assess the reliability of the tree. A nucleotide sequence of *E. bieneusi* from a kangaroo (GenBank: KY706128) was used as outgroup in phylogenetic analysis.

The statistical analysis was performed using the Statistical Package for the Social Sciences (SPSS) 19.0. Pearson chi-square (χ^2^) and Fisher’s exact tests were used to determine the relationship between *E. bieneusi* infection and possible risk factor variables and gastrointestinal symptoms (Table 2), respectively. The level of statistical significance was set as *P* < 0.05.

## Data Availability

ITS sequences of four novel *E. bieneusi* genotypes obtained in the present study were deposited in the GenBank database under the following accession numbers: MN399816–MN399819.

## Abbreviations

AIDS: Acquired immune deficiency syndrome
CI: Confidence interval
HIV: Human immunodeficiency virus
ITS: Internal transcribed spacer
OR: Odds ratio
PCR: Polymerase chain reaction
rRNA: Ribosomal RNA
